# Modelling hospital outcome: problems with endogeneity

**DOI:** 10.1186/s12874-021-01251-8

**Published:** 2021-06-21

**Authors:** John L. Moran, John D. Santamaria, Graeme J. Duke, The Australian & New Zealand Intensive Care Society (ANZICS) Centre for Outcomes & Resource Evaluation (CORE)

**Affiliations:** 1 Department of Intensive Care Medicine, The Queen Elizabeth Hospital, Woodville, Australia; 2 Department of Critical Care Medicine, St Vincent’s Hospital (Melbourne), Fitzroy, Australia; 3 Intensive Services, Eastern Health, Box Hill, Australia

**Keywords:** Outcome analysis, Logit, Probit, Linear probability model, Calibration, Endogeneity, Marginal effects

## Abstract

**Background:**

Mortality modelling in the critical care paradigm traditionally uses logistic regression, despite the availability of estimators commonly used in alternate disciplines. Little attention has been paid to covariate endogeneity and the status of non-randomized treatment assignment. Using a large registry database, various binary outcome modelling strategies and methods to account for covariate endogeneity were explored.

**Methods:**

Patient mortality data was sourced from the Australian & New Zealand Intensive Society Adult Patient Database for 2016. Hospital mortality was modelled using logistic, probit and linear probability (LPM) models with intensive care (ICU) providers as fixed (FE) and random (RE) effects. Model comparison entailed indices of discrimination and calibration, information criteria (AIC and BIC) and binned residual analysis. Suspect covariate and ventilation treatment assignment endogeneity was identified by correlation between predictor variable and hospital mortality error terms, using the Stata™ “eprobit” estimator. Marginal effects were used to demonstrate effect estimate differences between probit and “eprobit” models.

**Results:**

The cohort comprised 92,693 patients from 124 intensive care units (ICU) in calendar year 2016. Patients mean age was 61.8 (SD 17.5) years, 41.6% were female and APACHE III severity of illness score 54.5(25.6); 43.7% were ventilated. Of the models considered in predicting hospital mortality, logistic regression (with or without ICU FE) and RE logistic regression dominated, more so the latter using information criteria indices. The LPM suffered from many predictions outside the unit [0,1] interval and both poor discrimination and calibration. Error terms of hospital length of stay, an independent risk of death score and ventilation status were correlated with the mortality error term. Marked differences in the ventilation mortality marginal effect was demonstrated between the probit and the "eprobit" models which were scenario dependent. Endogeneity was not demonstrated for the APACHE III score.

**Conclusions:**

Logistic regression accounting for provider effects was the preferred estimator for hospital mortality modelling. Endogeneity of covariates and treatment variables may be identified using appropriate modelling, but failure to do so yields problematic effect estimates.

**Supplementary Information:**

The online version contains supplementary material available at 10.1186/s12874-021-01251-8.

## Background

Modelling mortality outcome has been a constant preoccupation within the critical care literature [1] both in terms of predictive models such as the Acute Physiology and Chronic Health Evaluation (APACHE) algorithms [2, 3] and ground up exploratory studies of the impact of covariates of interest [4]. The preferred model has been logistic regression (or logit) [5], rather than probit [6], consistent with the sentiments of Berkson, “Why I prefer logits to probits”, expressed 70 years ago [7]. In econometrics, the probit [8] and the linear probability model (LPM) [9] have been extensively used for modelling binary outcomes and such models have occasionally appeared in the biomedical literature [10].

Model validation has also differed between disciplines. Within the biomedical and epidemiological literature extensive discussion has focused around concepts of discrimination and calibration [11–13], whereas in econometrics bias and parameter consistency have been dominant [14–16], to the exclusion of model performance issues such as goodness-of-fit [17], although some issues intersect [18]. Econometrics has paid greater attention to concepts such as endogeneity [19], self-selection [20] and non-randomized treatment assignment [21], although there has been a rapid increase in the biomedical literature devoted to these issues, especially in epidemiology [22]. Previous attention [23] has been drawn to suspected endogeneity in mortality models where length of stay [24] or mortality probability [25] were entered as predictive covariates; such regression of a variable upon its components has been termed a “dubious practice” [26].

The purpose of this study was to explore the performance of regression models, logistic, probit and the LPM in predicting the hospital mortality risk of a large cohort of critically ill intensive care patients whose data was recorded in the ANZICS (Australian and New Zealand Intensive Care Society) Adult Patient Data Base [27]. Machine learning approaches were not considered [28], albeit there is debate as to what constitutes “machine learning” [29, 30]. Performance of both fixed and random effects models of logit and probit was compared with particular attention directed to calibration [13]. The following issues were also canvassed; the potential endogeneity of hospital length of stay (HLOS) and hospital mortality probability (ROD) recorded in the data base and derived from an independent published algorithm [31], and the effect of mechanical ventilation (MV) status (recorded as a binary variable) as an endogenous treatment assignment.

## Methods

### Ethics statement

Access to the data was granted by the Australian and New Zealand Intensive Care Society (ANZICS) Centre for Outcomes & Resource Evaluation (CORE) Management Committee in accordance with standing protocols; local hospital (The Queen Elizabeth Hospital) Ethics of Research Committee waived the need for patient consent to use their data in this study. The dataset was anonymized before release to the authors by ANZICS CORE custodians of the database. The dataset is the property of the ANZICS CORE and contributing ICUs and is not in the public domain. Access to the data by researchers, submitting ICUs, jurisdictional funding bodies and other interested parties is obtained under specific conditions and upon written request (“ANZICS CORE Data Access and Publication Policy.pdf”, http://www.anzics.com.au/Downloads/ANZICS%20CORE%20Data%20Access%20and%20Publication%20Policy%20July%202017.pdf).

### Data management

Data was accessed from the ANZICS Adult Patient Database [27]; in this instance for calendar year 2016 and processed as previously described in detail [32].

### Statistical analysis

#### Predictive models

To predict hospital mortality a base parsimonious logistic model (Logit1) was developed with a core set of predictor variables and their interactions, similar to previous papers utilizing data from the ANZICS Adult Patient Database [23, 32]; no automated routine for covariate selection, such as stepwise regression, was used. The covariate set was then supplemented by addition of two covariates: log HLOS (in days) and log risk of death (ROD) derived from a locally validated mortality algorithm (Australian and New Zealand Risk of Death model) [31] and model fit was further ascertained. All continuous variables were centred to improve model convergence. Using the same base covariate set and additions, this process was repeated for the following models:

1. Logistic regression with intensive care unit (ICU) providers as fixed effects (FE), (Logit2)
2. A base probit regression (base: Probit1)
3. Probit regression with intensive care unit (ICU) providers as FE (Probit2)
4. Random effects (RE) logit (Logit3) and probit regression (Probit3) with patients nested within ICU providers considered as RE; that is a random intercept model.
  - The intra-class correlation (ICC), the correlation between patients in ICU providers [33], was calculated for the null model (unconditional) and the full model (conditional) [34].
5. A base LPM (LPM1), and with ICU providers as FE (LPM2)
  - For the LPM, predictions were constrained within [0,1] using the linear discriminate function as suggested by Haggstrom [35, 36]: the LPM was estimated by ordinary least squares regression (OLS); the parameters were transformed (multiplied by *K* = *N*/*RSS*, where *N* is sample size, *RSS* is the residual sum of squares and *K* is > > 1); predicted probabilities were then generated using logistic regression [37]. The user written Stata command “reg2logit” [38] was utilised. Model indices were provided for this model (“LPM_ldm”) and for the vanilla linear regression model with probabilities constrained to the [0,1] range (“LPM [0,1]”).
6. Where of interest, predicted mortality probabilities were compared graphically using a limits of agreement (LOA) method, whereby the mean difference and the data were presented as paired differences plotted against pair-wise means. The user written Stata module “concord” was employed [39].

Model performance was assessed thus:

1. The traditional criteria of discrimination (receiver operator characteristic curve area, AUC) and calibration (Hosmer-Lemeshow (H-L) statistic). Although the H-L statistic will invariably be significant (*P* < 0.1 and H-L statistic > 15.99) in the presence of large N and increments to the grouping number (default = 10) of the H-L test have been recommended [40], the default grouping number was used.
  - Calibration plots (observed binary responses versus predicted probabilities) were undertaken using the user-written Stata module “calibrationbelt” [41]. The relationship of predictions to the true probabilities of the event was formulated with a second logit regression model, based upon a polynomial transformation of the predictions, the degree of the polynomial (beginning with second order) being forwardly selected on the basis of a sequence of likelihood ratio tests. The deviation of the calibration belt from the line of identity is indicated by the reported *P* value (< 0.05).
2. The potential for overfitting, or shrinkage statistics (determined by in-sample and out-of-sample predictive bias and overfitting, expressed in percentages) was undertaken using the user-written Stata module “overfit” [18, 42]; that is, a focus on predictive calibration. Ten-fold cross-validation with 500 repeated iterations were used.
  - Under conditions of non-applicability of the algorithm, a more traditional approach was used; development and validation model data sets were generated and various indices were generated on each data set using the user-written Stata module “pmcalplot” [43]: calibration-in-the-large [44], calibration slope, C-statistic for model discrimination and ratio of expected and observed events.
3. Model residual analysis was undertaken using the “binned residual” approach as recommended by Gelman and Hill [45] and implemented in Stata by Kasca [46]: the data was divided into categories (bins) based upon the fitted values and the average residual (observed minus expected value) versus the average fitted value was plotted for each bin; the boundary lines, computed as $2\sqrt{p\left(1-p\right)/n}$ where *n* was the number of points per bin, indicated ±2SE bounds, within which one would expect about 95% of the binned residuals to fall.
4. Model comparison was also undertaken by the Akaike Information Criterion (AIC), with the Bayesian Information Criterion (BIC) for non-nested models; lower values being optimal [47].
5. In view of the burgeoning literature on coefficient comparison between nested and non-nested non-linear probability models [48–50], we undertook full (*X-Y*) standardisation of logistic, probit and LPM (for both the full sample “LPM (all N)” and LPM [0,1]) coefficients using the “listcoef” Stata user-written command [51, 52]. Graphical display of the standardised coefficients utilised violin plots [53]: box plots incorporating estimated kernel density information via the user-written Stata command “vioplot” [54].
6. The Stata™ command “margins” was used to frame predictions under various scenarios [55, 56]; mortality effect over variables such as MV status was generated with due note of the overlapping 95% CI conundrum that such overlapping does not necessarily indicate lack of statistical difference [57, 58]. Although the analyses were performed using Stata ™ statistical software, similar functionality is provided in R statistical software [59].

#### Covariate endogeneity and selection bias

Endogeneity arises when there is a correlation between an explanatory variable and the regression error term(s), either in OLS (ordinary least squares) regression [60, 61] or probit and logit [62]; the causes being omitted variables, simultaneity (contemporaneous or past) and measurement error [60]. The paradigmatic econometric model is the Heckman selection model [63]. The consequences of non-random assignment and (self) selection bias, in terms effect estimate bias, have also been well documented in the biomedical literature [64–66]. Predictor variable endogeneity and the impact of endogenous treatment assignment were formally addressed utilizing the Extended Regression (ERM; “eprobit”) module of Stata™ statistical software [67]; in particular, the demonstration of a significant correlation between the error term of the variable in question and the error term of the dependent variable, hospital mortality. The method used by “eprobit” was to apply instrumental variables (IV), [68, 69]) which predict the endogenous variable(s) and have an outcome (mortality) effect via these endogenous variables [70], with robust standard errors (“vce (robust”) as recommended [71]. A third (unverifiable) assumption is that the IV-outcome association is unconfounded [72]. Using a potential outcomes scenario, the ventilation average treatment effect (ATE) and the average treatment effect of the treated (ATET, the mortality of those ventilated as opposed to the counterfactual mortality of these ventilated patients considered to be not ventilated) were estimated. Again, the “margins” command, suitably specified for “eprobit”, was used to estimate various scenarios on the probability scale; in particular, comparisons were “fixed” such that for endogenous treatment assignment patients were compared assuming all were ventilated and then all were not-ventilated (a counter-factual scenario). The variance-covariance matrix was specified as “unconditional” [73]; that is, via the linearization method, non-fixed covariates were treated in a way that accounted for their having been sampled, allowing for heteroskedasticity or other violations of distributional assumptions and for correlation among the observations in the same manner as vce (robust).

Stata™ Version 16.1 was used for all analyses and statistical significance was ascribed at *P* < 0.05. For continuous variables, results are presented as mean (SD) unless otherwise indicated.

## Results

### Cohort description

The cohort comprised 92,693 patients from 124 intensive care units (ICU) in calendar year 2016; 17% of ICUs were metropolitan (non-tertiary), 32.5% in private, 6.5% rural / regional and 44% were tertiary, as defined in the ANZICS-APD data dictionary [74]. Patient mean age was 61.8 (SD 17.5) years, 41.6%.

were female and APACHE III score 54.5(SD 25.6); 43.7% were ventilated. ICU length of stay was 3.1(SD 4.5) days and HLOS was 11.8(SD 13.0) days. ICU and hospital mortality were 6.45% (95%CI: 6.30, 6.61) and 8.82% (95%CI: 8.64, 9.00) respectively.

### Model performance: logit, probit and LPM

For the base logit (Additional file 1), probit and LPM_ldm models, the number of parameters at 110 was large but the shrinkage and overfitting indices did not indicate problematic overfitting (Table 1).

**Table 2:** Model performance for logit with addition of log HLOS and ROD (NC: not computed)

Model title	Logit1: lnday	Logit1: lnrod	Logit2: lnday	Logit2: lnrod	Logit3:lnday	Logit3:lnrod
Index						
ROC AUC	0.921	0.934	0.923	0.936	0.917	0.935
H-L statistic; P-value	0.000	0.060	0.000	0.013	0.000	0.054
Out-of-sample shrinkage %	0.940	0.020	−1.370	0.750		
In-sample-shrinkage %	0.380	−0.500	−2.350	−0.380		
Overfitting %	0.560	0.510	0.960	1.120		
Calibration belt: P-value	0.000	NC	0.000	0.000	0.000	0.000
AIC	31,306.41	30,574.13	31,077.06	30,492.24	31,148.98	30,523.52
BIC	322,421.1	31,489.52	33,039.96	32,455.96	32,073.81	31,448.36
Development set						
CITL					0.005	0.017
C-slope					1.005	1.004
AUC					0.923	0.935
E:O ratio					0.997	0.998
Validation set						
CITL					0.005	0.017
C-slope					1.005	1.004
AUC					0.923	0.935
E:O ratio					0.997	0.990

**Fig. 1:**
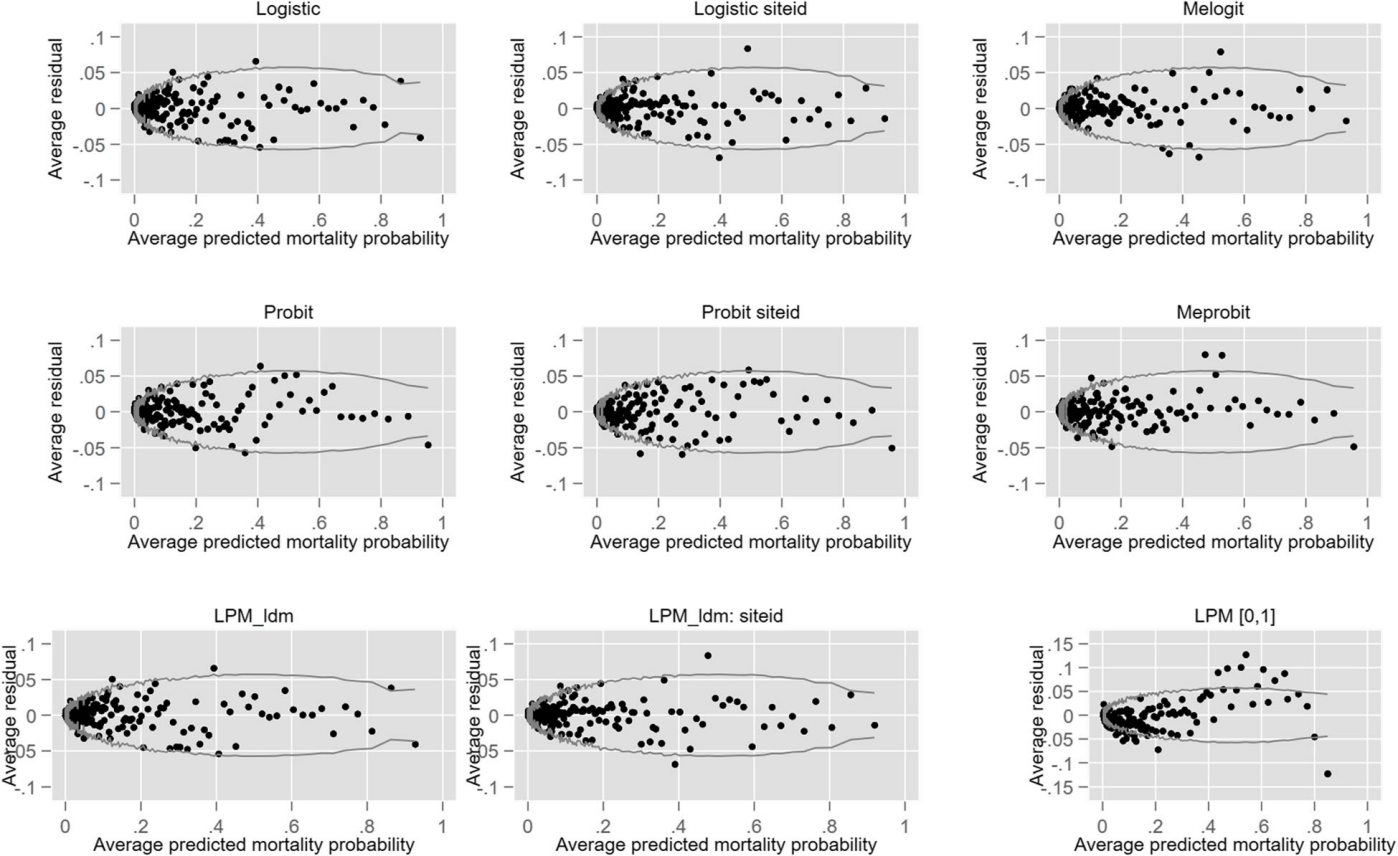
Binned residual graphs for logistic, probit and LPM models

Similarly, the use of ICU providers as FE substantially increased the number of parameters but again there was no evidence of overfitting, although the specification of the FE logit model (Logit2) dominated the FE probit (Probit2). The “overid” module was not applicable to the RE models and a more conventional development / validation data set approach was undertaken; the RE logit model had superior performance, at least by information criteria (BIC). The unconditional ICC in the RE logit model was 0.201 indicating a modest patient correlation within ICUs; not surprisingly, the conditional ICC decreased to 0.018. Patient number for the LPM [0,1] model it was 68,264 as the generated probabilities were < 0 in 24,179 (35.4%) and > 1 in 250 (0.4%). There was little difference in the pattern of the residual graphs between the eight models, except for the vanilla probit model where there was more asymmetry about the null (zero) line as seen in Fig. 1.

**Fig. 2:**
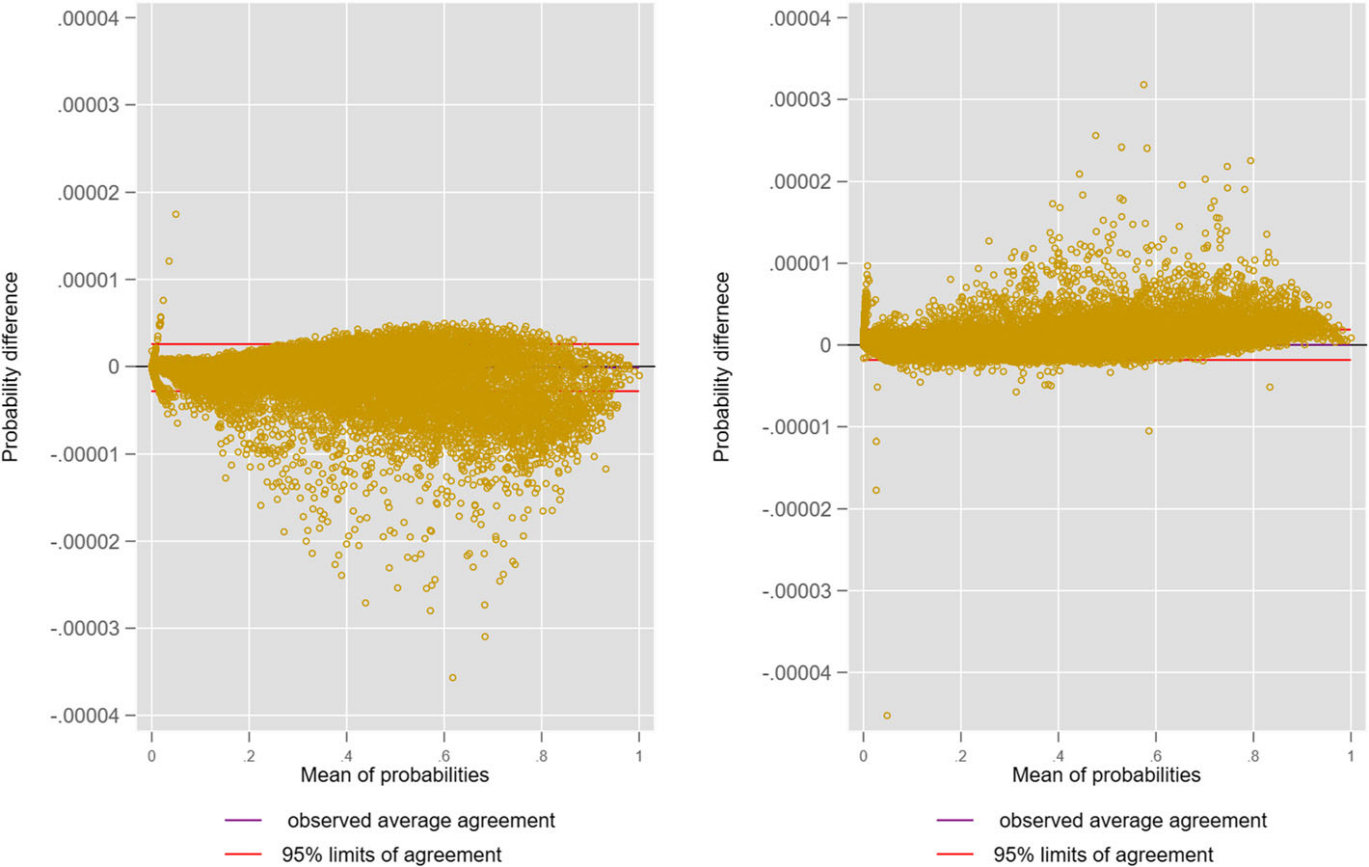
LOA between logistic and LPM_ldm probabilities:left, base models; right ICU providers as FE (*N* = 92,693)

Despite having a satisfactory discrimination (AUC: 0.884), the LPM [0,1] model demonstrated poor calibration as displayed by the lack of fit in the residual analysis (Fig. 1). The predicted probabilities of the vanilla logistic and LPM_ldm models were of some interest and were illustrated using a LOA graph (Fig. 2); the differences were exceptionally small, although in opposite directions for vanilla models versus those with ICU provider FE.

### Model effect of potentially endogenous covariates

**Fig. 3:**
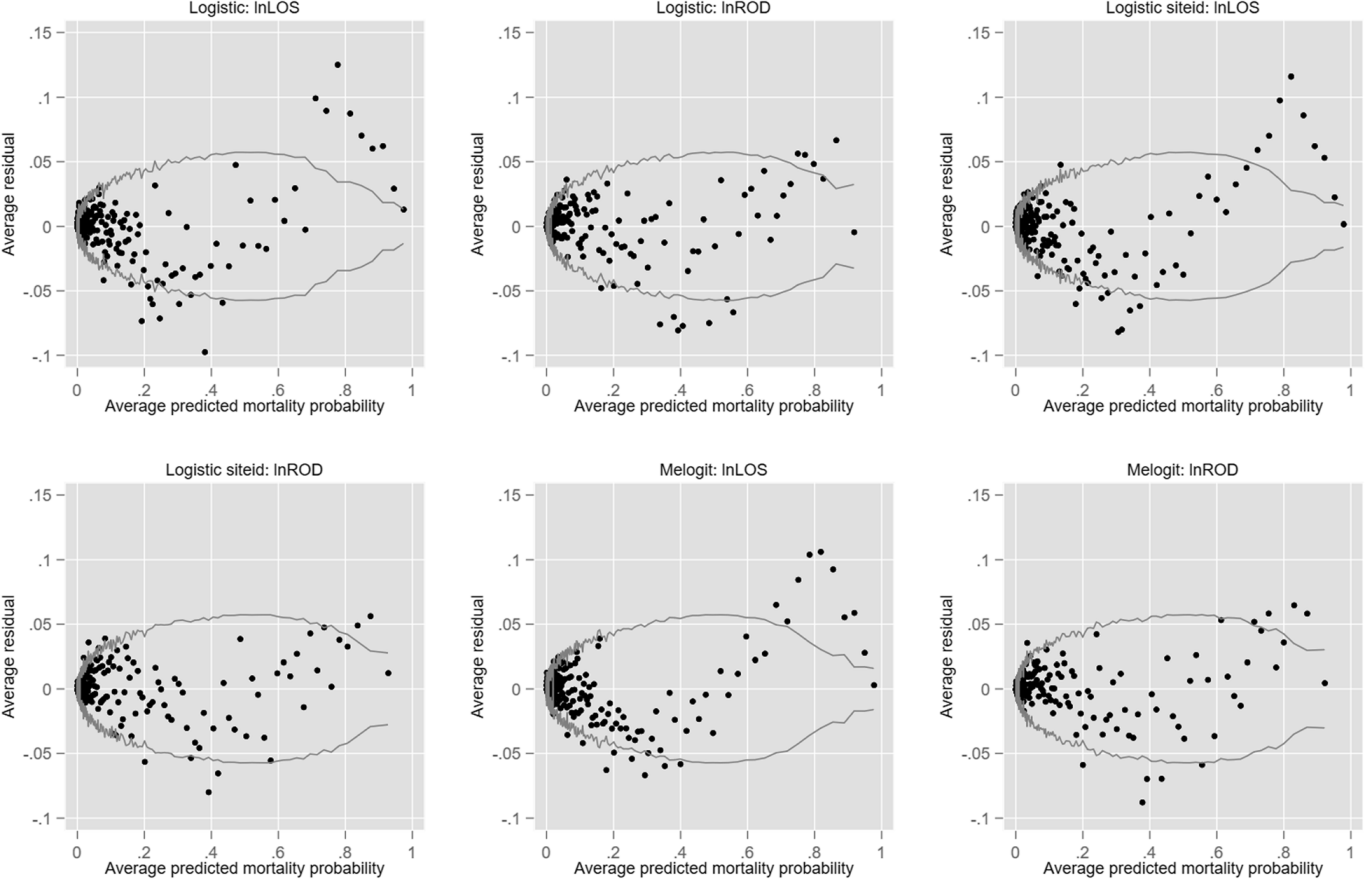
Binned residual graphs for logistic models with added variables (log) HLOS and ROD

As consideration was also given (see below) to the impact of two potentially endogenous covariates (HLOS and a ROD score), both in log form, a summary of the effect of the addition of these two covariates upon model performance for logit (Logit1, Logit2 and Logit3) is presented in Table 2 and Fig. 3.

**Table 1:** Model performance estimates for the base models

Model title	Logit1	Logit2	Probit1	Probit2	Logit3	Probit3	LPM1	LPM2	LPM0
Regression method	Logisitc	Logit1 + site FE	Probit	Probit1 + site FE	Logit 2 + RE	Probit2+RE	LPM_ldm	LPM_ldm+siteFE	LPM[0,1]
Indices									
Number of patients	92,693	92,693	92,693	92,693	92,693	92,693	92,693	92,716	68,264
Number of parameters	110	234	110	234	107	107	110	234	110
ROC AUC	0.915	0.917	0.915	0.917	0.917	0.917	0.912	0.915	0.884
H-L statistic; *P*-value	0.173	0.044	0.000	0.000	0.073	0.000	0.173	0.103	0.000
Out-of-sample shrinkage %	0.940	1.600	0.940	0.580			0.390	0.360	
In-sample-shrinkage %	0.380	0.360	0.380	−0.510			0.000	0.360	
Overfitting %	0.560	1.250	0.560	1.090			0.390	0.000	
Calibration belt: *P*-value	0.850	0.733	0.000	0.000	0.593	0.000	0.850	0.987	0.000
AIC	33,867.39	33,712.31	33,897.66	33,756.53	33,758.82	33,799.85	33,863.39	33,712.31	
BIC	34,792.25	35,665.78	34,803.62	35,710	34,674.21	34,715.24	34,769.35	35,665.78	
Development set									
CITL					−0.002	0.000			
C-slope					1.005	1.009			
AUC					0.917	0.915			
E:O ratio					1.001	1.000			
Validation set									
CITL					0.002	0.021			
C-slope					1.005	1.006			
AUC					0.916	0.916			
E:O ratio					0.989	0.987			
ICC: unconditional					0.201	0.154			
ICC: conditional					0.018	0.016			
ICC: unconditional					0.201	0.154			
ICC: conditional					0.018	0.016

Small increments in the AUC and decrements in both AIC and BIC were seen for all logit models compared with the base models (Table 2). There was, however, substantial loss of model calibration and disturbances in residual distribution, more so for the addition to the base model of HLOS than for ROD.

### Model coefficients

**Fig. 4:**
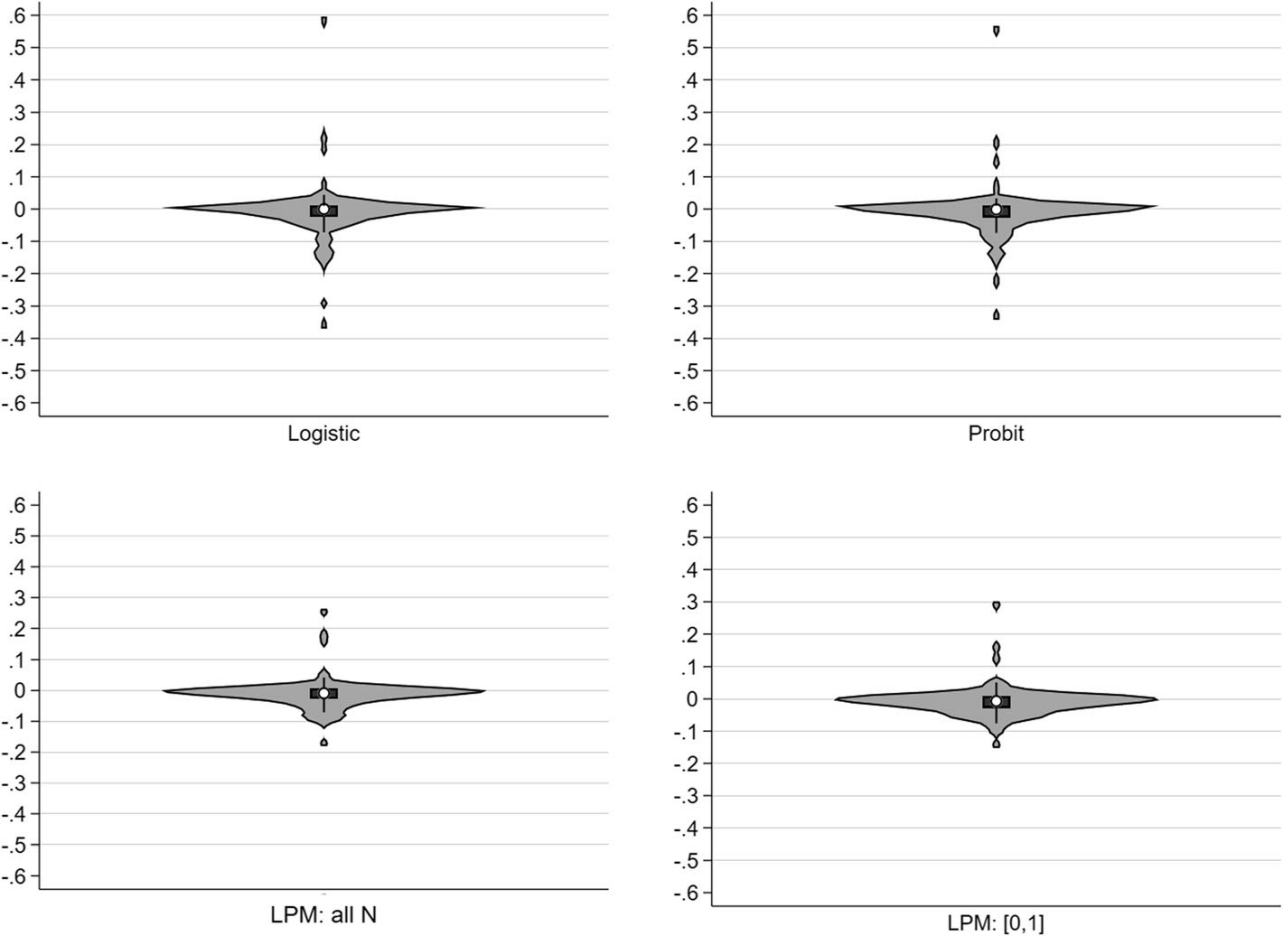
Violin plots of model coefficients for logistic, probit and LPM models

For the models Logit1, Probit1, LPM (all N) and LPM0, using robust SE [71], the fully standardised β coefficients are seen in Fig. 4. The LPM_ldm model (LPM1) was not used as the β coefficients were transformed.

There was moderate conformity between the density distribution of the four models, but this belied a quantitative comparison using simple regression of the scalar values of the full (*X-Y*) standardised β coefficients (*n* = 100) with logistic as the comparator (Table 3).

**Table 3:** Standardised β coefficients, logit versus probit and LPM

Logit	β	*P*-value	95%CI: lower	95%CI: upper
Probit	1.204	0.000	1.186	1.221
LPM: all N	0.028	0.536	−0.061	0.116
LPM: [0,1]	−0.301	0.000	−0.400	−0.202

There was a sizeable overall difference between the average scalar β model coefficients. Of interest, the number of model significant coefficients was 50 in the logit, 55 in the probit, 63 in the LPM (all N) and 69 in the LPM0.

#### Endogeneity and non-random assignment

##### APACHE III severity of illness score

As the APACHE III score [2] was a key variable measuring patient severity of illness, the status of this variable with respect to endogeneity was tested using age, hospital level (4 level categorical variable) and APACHE III diagnosis (categorical variable denoting 28 collapsed APACHE III diagnostic codes; see Additional file 1) as IV. There was no evidence for endogeneity; error correlation of APACHE III score v mortality outcome: 0.000(− 1.000, 1.000, *p* = 1.0000).

#### Endogenous covariates

Models with both risk of death and HLOS as endogenous variables failed to converge and the use of ICU providers as instruments failed to yield marginal estimates after 36 h of computation. The attempt to estimate the MV effect over the span of HLOS and risk of death using margins was also unsuccessful due a nonsymmetric or highly singular matrix. For log HLOS and log ROD as endogenous variables, and MV status as an endogenous treatment assignment, the best model by information criteria used the APACHE III score, hospital level, APACHE III diagnostic categories and annual patient volume (as deciles) as IV, with a substantial reduction (up to 13%) of both AIC and BIC compared with models using a lesser number of IV.

There was a significant correlation between the error terms of the dependent variable (hospital mortality) and both ventilation status and log HLOS, and between ventilation status and log HLOS, as seen in Table 4. The ATE and ATET were 5.38% (95%CI: 1.33, 9.44) and 4.55% (95%CI: 0.98, 8.13) respectively. For a comparable probit model with log HLOS added as an extra covariate (using the “margins” command), the ventilation mortality effect was 0.48 (95%CI: 0.10, 0.85).

**Table 4:** Correlation of model error terms “.e” for mortality, ventilation and HLOS

Correlation	Estimate	Robust SE	z-value	*p*-value	95%CI_lower	95%CI_upper
Ventilation.e vs mortality.e	−0.248	0.085	−2.93	0.003	−0.405	−0.076
Log HLOS.e vs mortality.e	−0.315	0.007	−45.50	0.000	−0.328	−0.301
Log HLOS.e vs ventilation.e	0.119	0.005	24.56	0.000	0.109	0.128

#### Mechanical ventilation effect

##### Log HLOS

**Fig. 5:**
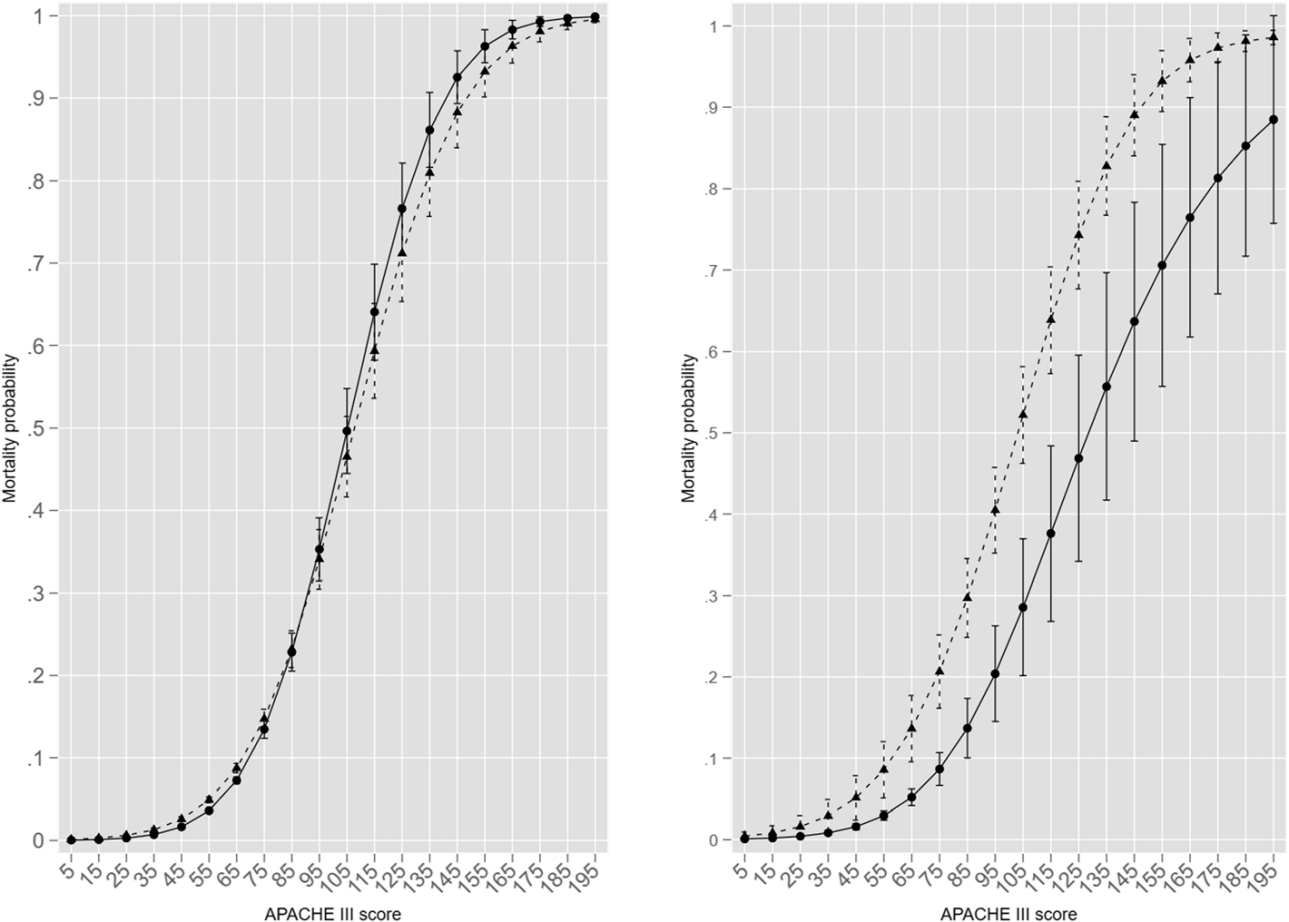
Mortality MV effect over the span of the APACHE III score with the log HLOS modelled as an endogenous variable for probit model on left, "eprobit" (HLOS endogenous) on right. MV effect as black triangles circles and non-ventilation as solid black circles with 95%CI

The mortality MV effect over the span of the APACHE III score is shown in Fig. 5 with the log HLOS modelled as an endogenous variable; the comparator is the probit model with log HLOS as an added covariate to the base model. There is an apparent mortality increment across high APACHE III scores for non-ventilation in the probit model, but this is reversed in the "eprobit" model.

**Fig. 6:**
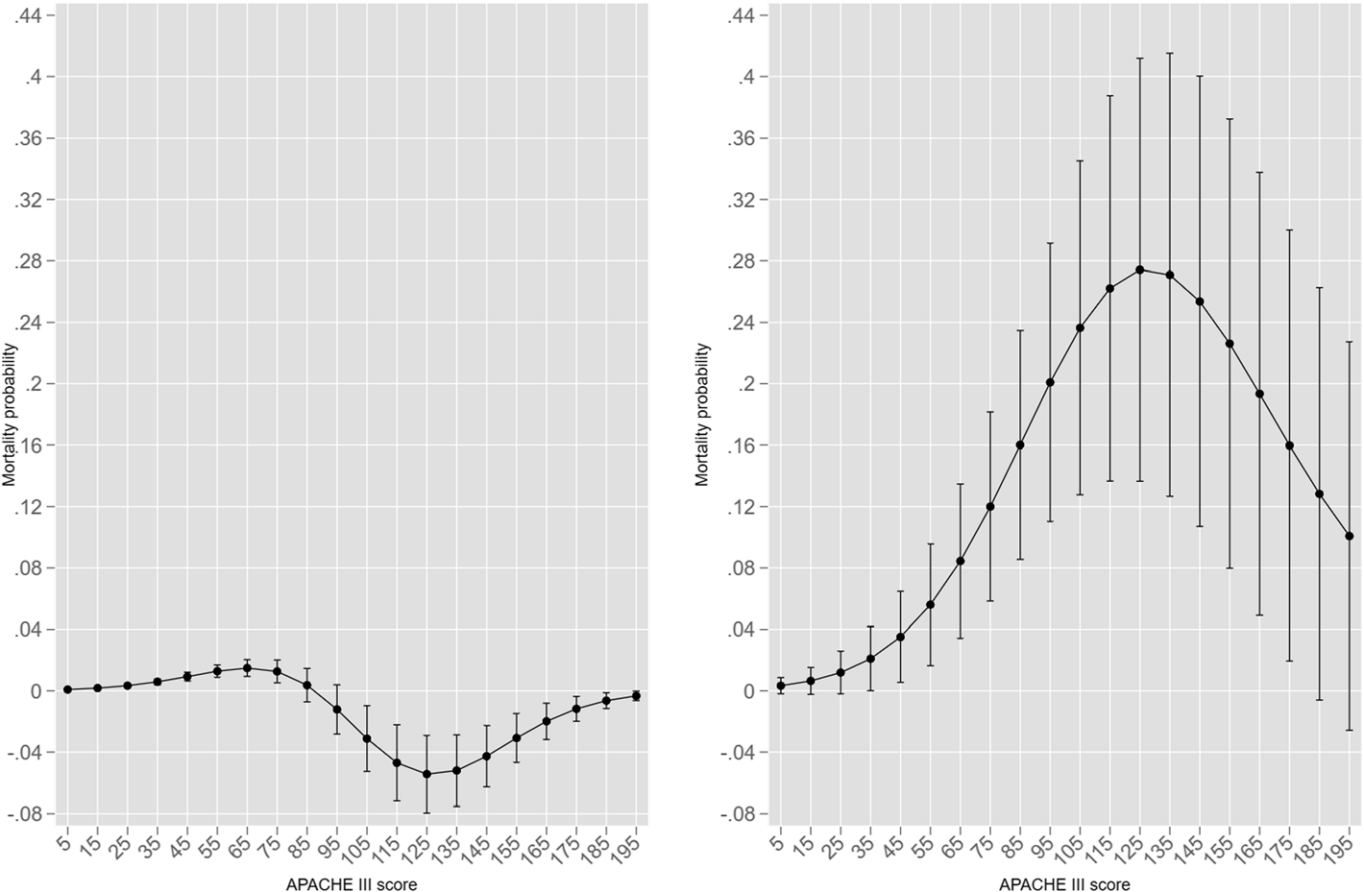
Ventilation mortality contrast (absolute difference, MV versus non-ventilated, y-axis: ± about the null difference of 0) for probit model on left, "eprobit" (HLOS endogenous) on right. MV contrast effect (ventilated versus not-ventilated) as solid black circles with 95%CI

The ventilation mortality contrast (absolute difference, MV versus non-ventilated, y-axis: ± about the null difference of 0) for both models is seen in Fig. 6 and exhibits model differences with greater clarity. The mortality increment across high APACHE III scores (range 105–175) for non-ventilation in the probit model reached statistical significance but was quite small. The mortality contrast in the eprobit model was substantial across almost the entire range of APACHE III scores 5–175.

##### Log ROD

For the log risk of death score modelled as an endogenous variable there was significant correlation between the error terms of the dependent model variable (hospital mortality) and both MV status and log risk of death, and between MV status and log risk of death, as seen in Table 5. The ATE and ATET were 3.07% (95%CI: − 0.28, 6.43) and 2.95% (95%CI: − 0.35, 6.24) respectively. For a comparable probit model with log risk of death score added as an extra covariate (using the “margins” command), the MV mortality effect was − 0.58% (95%CI: − 0.93, − 0.23).

**Table 5:** Correlation of model error terms “.e” for mortality, ventilation and ROD

Correlation	Estimate	Robust SE	z	*p*-value	95%CI_lower	95%CI_upper
Ventilation.e vs mortality.e	−0.235	0.089	−2.640	0.008	−0.399	−0.055
Log ROD.e vs mortality.e	0.471	0.008	58.29	0.000	0.455	0.486
Log ROD.e vs ventilation.e	−0.052	0.005	−10.35	0.000	−0.062	−0.042

**Fig. 7:**
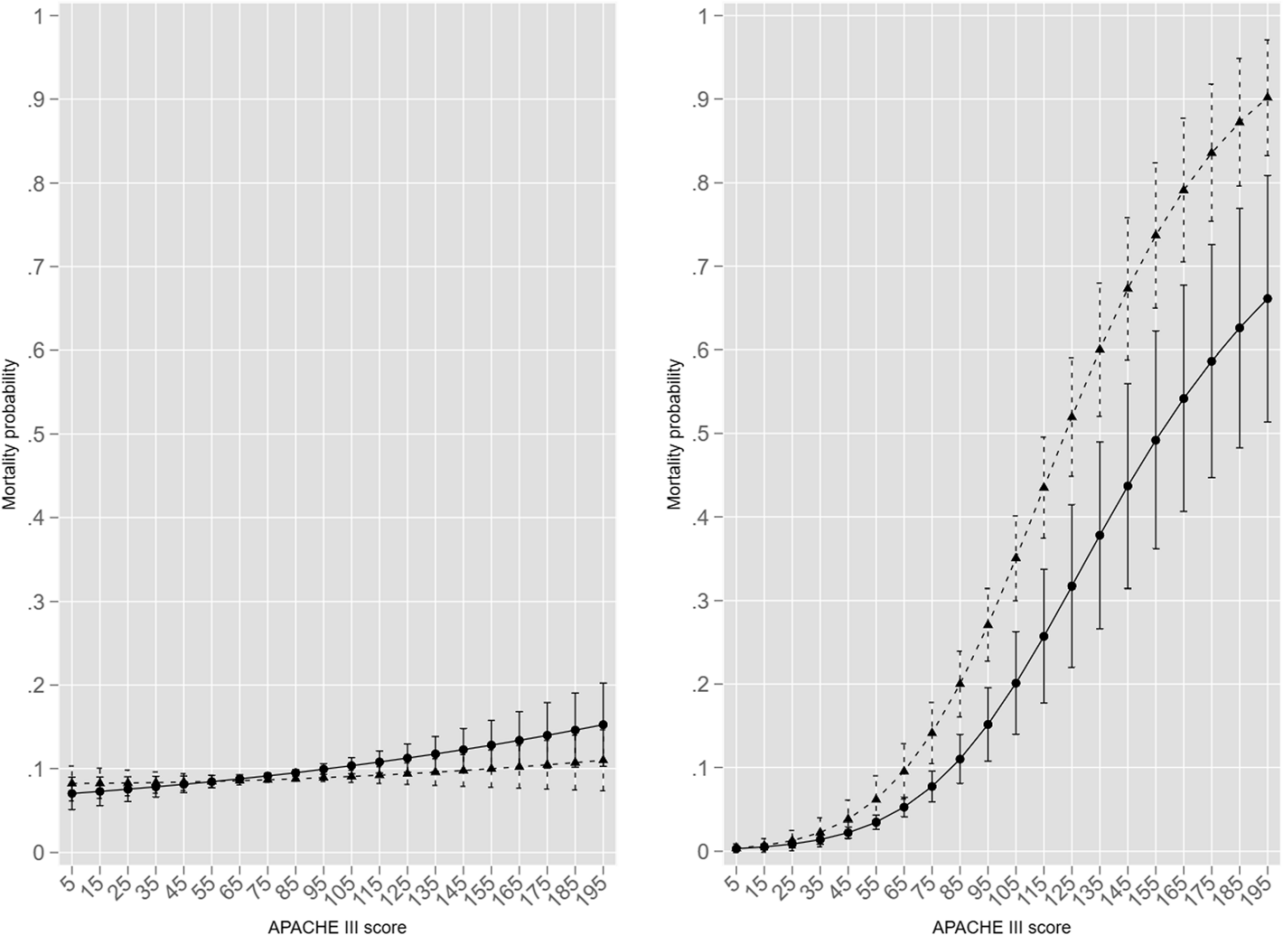
Mortality MV effect over the span of the APACHE III score with the log ROD modelled as an endogenous variable for probit model on left, "eprobit" (ROD endogenous) on right. MV effect as black triangles and non-ventilation as solid black circles with 95%CI

The mortality MV effect of the above ERM model is shown in Fig. 7 with the log risk of death modelled as an endogenous variable; the comparator is a probit model with log risk of death as an added covariate to the base model. There was an apparent differential mortality increment for non-ventilation versus MV in the probit model at an APACHE III score of 85, but this reversal was not apparent in the eprobit model. The overall mortality effect of the added variable log risk of death in the probit model was quite modest compared with that of log HLOS.

**Fig. 8:**
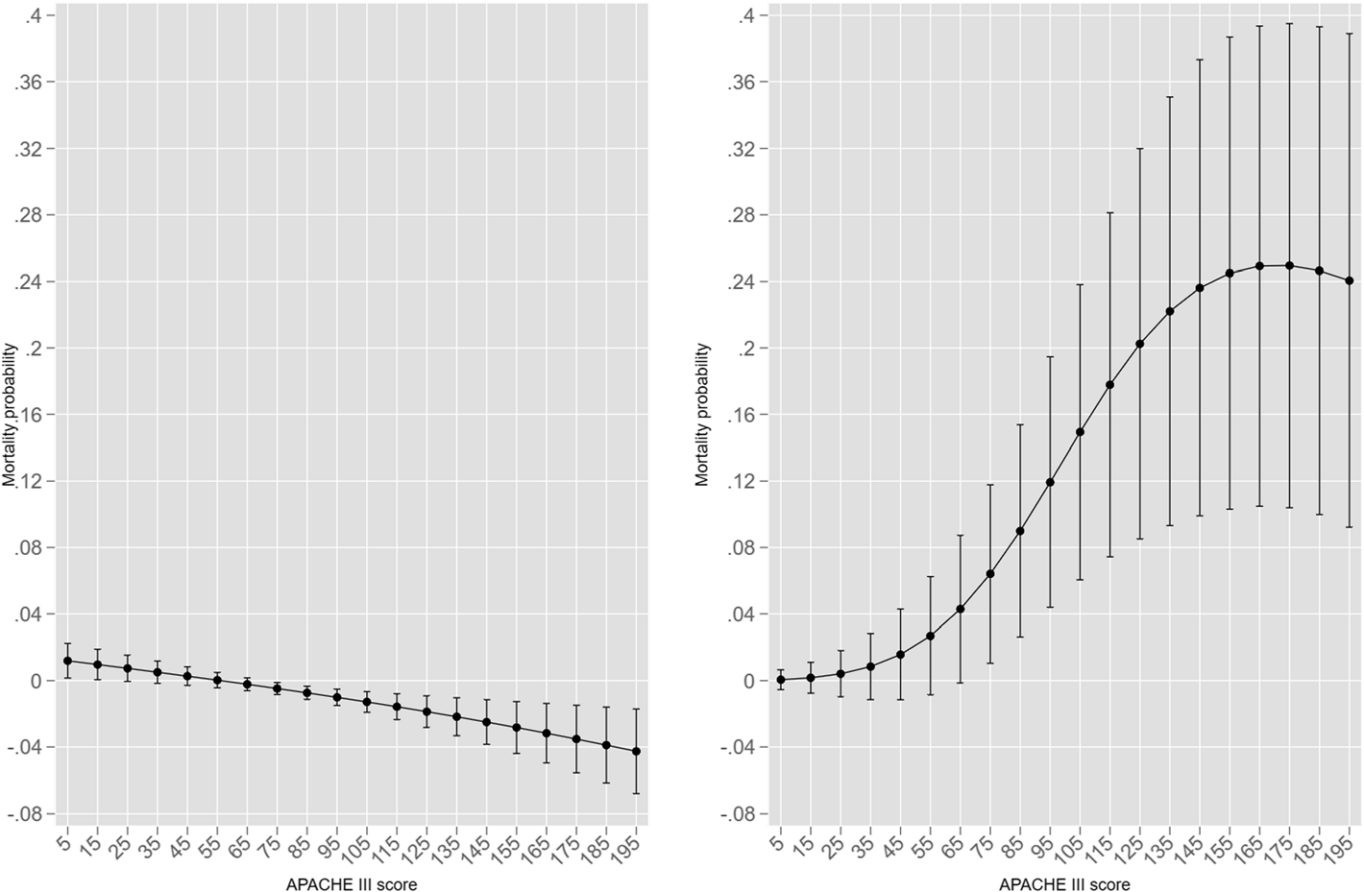
Ventilation mortality contrast (absolute difference, MV versus non-ventilated, y-axis: ± about the null difference of 0) for probit model on left, "eprobit" (ROD endogenous) on right. MV contrast effect (ventilated versus not-ventilated) as solid black circles with 95%CI

The MV mortality contrast (MV versus non-ventilated) for both models is seen in Fig. 8, and again demonstrated the difference with more transparency. For the probit model there was a small mortality increment for non-ventilation across APACHE III score 75–195, but this was not reflected in the eprobit model where a marked ventilation mortality increment occurred across APACHE III scores 85–195.

For the "eprobi"t graphic displays, 95%CI span was greater than that of the probit.

## Discussion

Of the eight models considered in predicting hospital mortality, logit regression (with or without ICU providers as FE) and RE logit dominated, more so using information criteria indices, in accordance with a recent extensive simulation study [75]. The LPM suffered from many predictions outside the unit interval, but the LPM_ldm model demonstrated, perhaps not surprisingly, a performance similar to that of the logit model. HLOS and the ROD score were demonstrated to be endogenous variables and patient ventilation status as an endogenous treatment assignment variable. Marked differences in the MV mortality effect was demonstrated between the vanilla probit and the eprobit models which were scenario dependent. These findings are further discussed.

### Logistic regression as the preferred estimator

In biomedicine binary data analysis invariably proceeds using logistic regression in its various forms. Vach notes that “… probit regression and logistic regression give very similar results with respect to the order of the magnitude of the effect estimates” [76]; that is, the familiar scalar multiplier: ${\hat{\beta}}_{\mathrm{Logit}}\simeq 1.6{\hat{\beta}}_{\mathrm{Probit}}$ [77]. This belies the demonstrated differences in the fully standardised (*X-Y*) coefficients of the logistic and probit models in the current study. That the OR is difficult to interpret and is mis-conceived as a RR [78] has become a mantra. However, the interpretation of the probit coefficient is not immediately apparent, being the difference in Z score associated with each one-unit difference in the predictor variable. More generally, it must be noted that the three popular indices of risk, OR, RR and risk difference (RD), are neither related monotonically nor are interchangeable and the “… results based upon one index are generally not translatable into any of the others” [79]. A substantial literature in the social sciences has addressed the problem of coefficient comparison across groups in non-linear probability models, probit and logit, on the basis of unobserved heterogeneity, beginning with the seminal 1999 paper of Allison [80]. We do not pursue this theme [81] further, rather, submit that coefficient non-concordance is a function of the well described non-collapsibility of both odds ratios and probit regression coefficients [56, 82] and may be suitably resolved using marginal effects, including effect derivatives, on the probability scale [16, 83]: “… the output from non-linear models must be converted into marginal effects to be useful. Marginal effects are the (average) changes in the CEF [conditional expectation function: the expectation, or population average, of *Y*_*i*_ (dependent variable) with *X*_*i*_ (covariate vector) held fixed] implied by a non-linear model. Without marginal effects, it’s hard to talk about the impact on observed dependent variables” [84].

Most models achieved an AUC of ≥0.9 with between model AUC differences being small; the lack of import of such small AUC differences has been canvassed [85]. The primacy of AUC [86] in model assessment, as in machine learning, would appear to be misplaced [87] and calibration indices should be fully incorporated into analysis [88]. Certainly, the graphic residual analysis provided an extra dimension in revealing the effect on model goodness-of-fit with the addition of the two suspect (see below) endogenous predictor variables, HLOS and ROD score (Table 1 and Fig. 2). The stability of the logit and probit FE estimation, with 123 extra parameters (Table 2 and Fig. 1), was reassuring. There has been considerable debate in both the econometric and statistical literature regarding performance (consistency) of the maximum likelihood estimator in the presence of FE, particularly large group numbers; the “incidental parameters problem” [89]; such concerns may be more apparent than real [90–93].

The choice between vanilla logistic regression (Logit1) and logistic regression with fixed site effects (or dummies [94], Logit 2) and random effects (Logit 3), would appear to depend upon purpose [95]. Transportable models, such as APACHE III [2] and the Australian and New Zealand Risk of Death model [31], eschew site fixed effects for logical reasons. The RE model is “sensible for modelling hierarchical data” [96], perhaps *de rigeur,* and with large data sets the computational demands of implementing, say, adaptive Gauss-Hermite quadrature, can be reasonably overcome by parallelisation, available in Stata™. The RE constraint of independence of provider effect (the random effects) from risk factors is often assumed, but it is plausible that such a correlation may “commonly occur” with consequent estimation bias [97]. Such constraint is not shared by high dimensional logistic FE models, which may have advantage [96], not the least of which is accounting for unobserved heterogeneity and, within the domain of profiling analysis, a smaller error for “exceptional” providers [96, 97]. Such conclusion was endorsed by Roessler et al. [98], who also noted the “sparse literature on fixed effects approaches”. Correlated RE models, for instance the Mundlak approach, are estimable for binary outcomes within the generalised linear mixed model framework (GLMM), as in the user-written Stata command “xthybrid” [99] and has been used in hospital outcome analysis [100]. Based upon our findings (Table 2 and Fig. 1), a probit RE model (Probit 3) had no advantage over the logit RE (Logit 3) and the inherent complexities of probit coefficient interpretation would not recommend it, albeit marginal effects on the probability scale are transparent. Moreover, the explained variance (McKelvey & Zavoina [101]) of the two RE models favoured the logit (${R}_{\mathrm{dichot}}^2$ = 0.62 (logit) versus 0.52 (probit)), where ${R}_{\mathrm{dichot}}^2=\frac{\sigma_F^2}{\sigma_F^2+{\tau}_0^2+{\sigma}_R^2}$; ${\sigma}_F^2$ is the linear predictor variance, ${\tau}_0^2$ is the intercept variance and ${\sigma}_R^2$ is the level one residual variance (fixed at *π*^2^/3 = 3.29 for the logit and 1 for the probit).

With respect to the profiling paradigm, which was not formally addressed, the contemporary choice between so-called “caterpillar plots” of provider effect estimates (plus 95% CI) [100] and funnel plots [102] would appear to have favoured the latter. The confidence intervals of the caterpillar plot “… are appropriate for testing single hypotheses … They are not appropriate for drawing inference about whether a given hospital’s performance is different from a set of their peers’ performances” [100]. This belies the difference between marginal and simultaneous confidence sets for ranks, whereby simultaneous confidence sets are robust to the latter inferential comparisons [103]. Such confidence sets for ranks have been implemented as “csranks” in both the Stata and R statistical environments.

The use of the LPM for binary data has generated controversy in the social science and econometric literature, but not in the biomedical; perhaps not surprisingly. However, these issues are addressed here. Firstly, a distinction must be made between the LPM as a preferred model versus its use as an alternative to logistic regression because of OR interpretational differences [104, 105]. We have alluded to this problem above, but it is disconcerting to find in a recent paper that the authors [104], whilst sympathetic to the average marginal effect (AME) as satisfying the criteria of comparability across both models and studies, quote the paper of Mood [56], published in 2010, to the effect that “deriving AME from logistic regression is just a complicated detour”. They conclude that “… we explore this procedure no further given its similarity to OLS results and the need for special-purpose routines to no notable advantage” and proceed to offer the LPM and a Poisson working model to compute risk differences and risk ratios, respectively. This ignores the fact that both risk differences and risk RR are collapsible metrics, as opposed to OR and probit coefficients. In Stata™, the “margins” command, introduced in Version 11 (July 2009), is a seamlessly integrated post-estimation tool, albeit it has undergone relevant computational revisions [16].

The question of bias and inconsistency of LPM estimates is somewhat moot: Horace and Oaxaca [14] argued from a theoretical perspective that the LPM was an inconsistent and biased estimator; simulation studies [49, 104, 105] suggest that LPM coefficient estimates were similar in magnitude and significance to that of logistic and probit regression but may be sensitive to continuous covariate distributional shape [106]. In finite examples, as in this study, such similarity was not fully achieved despite using robust standard errors to correct for LPM heteroscedasticity [49]; see Results. Model choice is properly determined by analytic purpose [9]. If outcome probability generation constrained to the unit interval is of importance, for instance the calculation of provider standardised mortality ratios, then the LPM cannot be endorsed, despite recommendations for prediction truncation, which may dramatically reduce study number, 26% in our data set, and converting continuous variables to factor levels [9, 48, 49, 54, 105]. The utility of a command such as “re2logit” for the purpose of generating probabilities from a LPM consonant with that of logit appears unclear. The method assumes multivariate normality which would not appear to be a fatal weakness [107, 108] and although complete or quasi-complete separation may occur with logistic regression [109, 110] and not with LPM, it was not observed in the current large N study [111]. Separation in logistic regression has been addressed from within the Social and Political Science domains in terms of advocacy for the LPM [94, 112] based upon estimation bias due to data omission. Under conditions of sparse data and separation alternate estimators are currently available, such as Firth’s penalised logit and the Mundlak correlated RE formulation which do not incur the prediction penalty of the LPM [94, 113–115]. There may be domain specific preference for the LPM: “… while a non-linear model may fit the CEF for LDVs [limited dependent variables; in this case, binary] more closely than a linear model, when it comes to marginal effects, this probably matters little” [84]. Where probability generation is required, “reg2logit” provides a useful addendum [37].

### Endogeneity

Endogeneity, as opposed to exogeneity, is conventionally ascribed to an explanatory variable (*x*), if the stochastic error (*ε*) in modelling the dependent variable (*y*) is not independent of *x*; that is, if E(*ε*| *x*) ≠ 0, then E(*y*| *x*, *ε*) ≠ E(*y*| *x*) [116]. The causes of endogeneity include omitted variables [63], measurement error, simultaneity (current or past), autocorrelated errors and sample selection [17]; the end result being biased and inconsistent estimates [70]. Endogeneity may occur in the presence [117] or absence of unobserved heterogeneity [118] and is to be distinguished from confounding; endogeneity articulated as “confounding by indication” would appear to be a contradiction [119]. Large sample size (‘big data”) does not limit the consequences of endogeneity [120, 121]. In the current analysis, where some of the effect of the error term(s) was attributed to the explanatory variable, the optimal course of action would be to “purge” the model of the correlation between the explanatory variable and the error term [19]; to wit, the use of the “eprobit” estimator.

Variables may be conceived by the analyst as endogenous, but it is not evident that in biomedical observational data analysis that particular attention has focused on the modelling consequences [120, 122]. The adverse effect of mechanical ventilation per se has been incorporated seamlessly into mortality prediction models without adjustment for patient selection; that is, a non-random physician treatment decision. The use of mortality probability as an independent variable in mortality prediction would appear to qualify as the regression of a variable upon its components [26]. Prolonged hospital length of stay is conventionally associated with mortality increment but displays a recursive effect or (current) simultaneity. With a large data set, it may not be obvious why the inclusion of one of the two endogenous covariates (HLOS or ROD score) should produce substantial loss of model calibration and disturbances in residual distribution; this may be a signal of an over-parameterised model and / or covariate endogeneity. We previously [123] demonstrated endogeneity of duration of mechanical ventilation in the critically ill ([123], Supplementary Appendix, figure E3) and performance of a tracheostomy as a non-random treatment variable, giving support to the notion that in the critical care domain, the effect of data variables realising complex patient-physician interaction may be endogenous. Similar studies have addressed the endogeneity of ICU admission decisions [124] and therapeutic titration based upon patient severity of illness [120, 121].

**Fig. 9:**
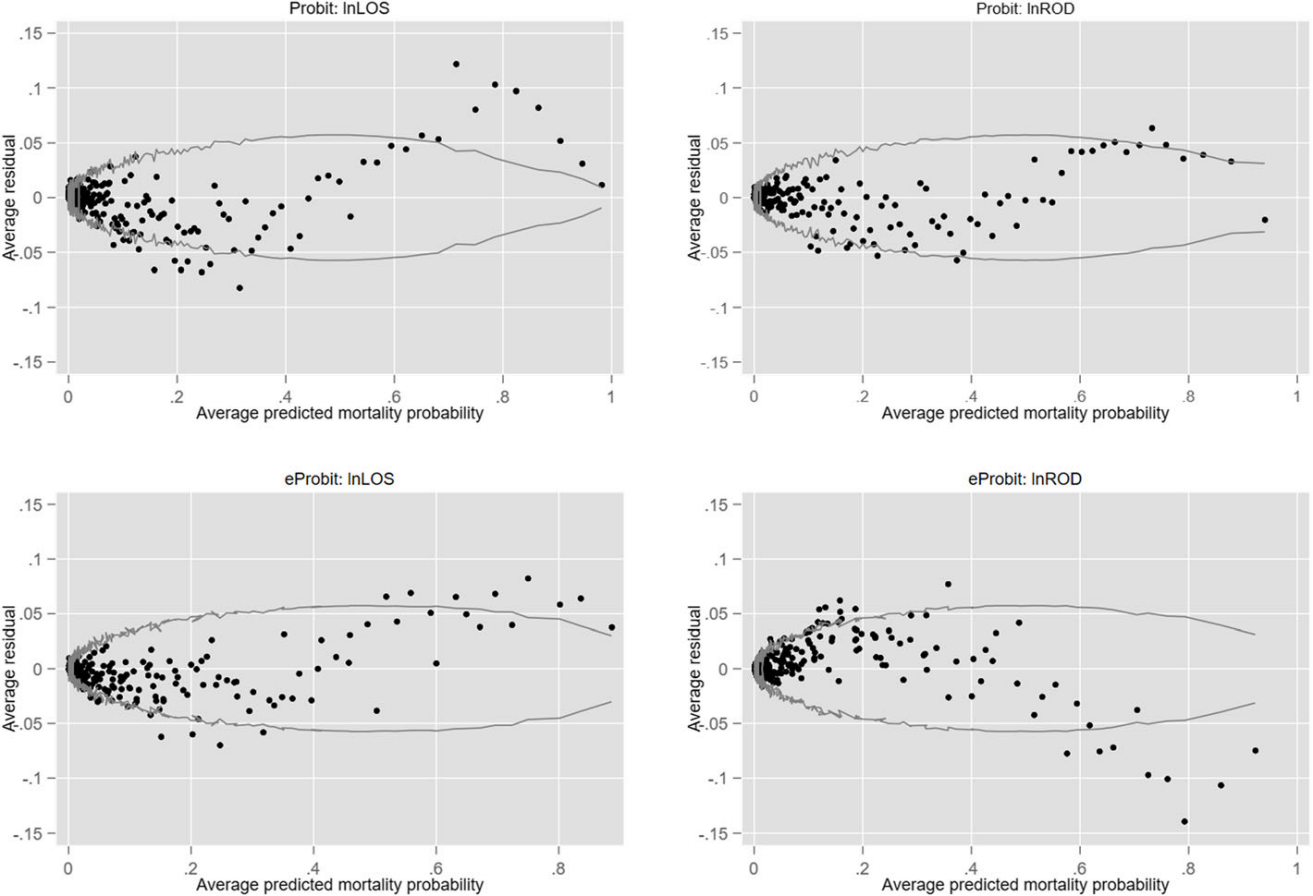
Model residual analysis: probit (upper panels) and “eprobit” (lower panels) for (log) HLOS and ROD as endogenous variables

Within the limits detailed in Results, substantial differences in both magnitude and direction of the ventilatory effect were demonstrated between the vanilla probit and the “eprobit” models by virtue of accounting for endogeneity. Contrast graphics also possessed merit in that they more clearly demonstrated effect differences obfuscated by seemingly overlapping 95% CI. The difference between the predicted marginal ventilation effects of vanilla probit and eprobit were not accompanied by any substantive improvement in model fit of “eprobit” versus probit and model residual analysis did not substantially favour “eprobit”, as seen in Fig. 9 (see also Duke and co-authors [123], Supplementary Appendix, figure E1 AND E2).

Similarly, model performance indices were not substantially different as seen in Table 6.

**Table 6:** Model performance indices, vanilla probit vs “eprobit”

Model	Probit:lnHLOS	Probit: lnROD	eProbit:lnHLOS	eProbit: lnROD
Index				
ROC AUC	0.921	0.934	0.923	0.930
H-L statistic; P-value	0.000	0.000	0.000	0.013
Calibration belt: *P*-value	0.000	0.000	0.001	0.001
CITL	−0.017	− 0.008	−0.050	0.059
C-slope	1.014	1.004	1.096	0.911
AUC	0.920	0.934	0.917	0.913
E:O ratio	1.010	1.004	1.033	0.967

Not surprisingly, the parameter coefficients for the two models were different, in magnitude and direction, as shown in Table 7 for lnROD considered as an endogenous variable. Estimates, as response derivatives (*dy*/*dx*), are displayed for main effects only.

**Table 7:** Main effects parameter coefficients (*dy*/*dx*) for probit and eprobit

	Probit				eProbit			
	dy/dx	*P*-value	95% CI (lower)	95% CI (upper)	dy/dx	*P*-value	95% CI (lower)	95% CI (upper)
Age_centered	0.0001	0.5810	−0.0002	0.0004	0.0001	0.3890	−0.0002	0.0004
APIII score_centered	0.0002	0.1570	−0.0001	0.0004	0.0022	0.0000	0.0020	0.0025
Gender								
Female	0.0000				0.0000			
Male	0.0001	0.9700	−0.0029	0.0030	0.0000	0.9810	− 0.0026	0.0026
AP III diagnostic codes								
Cardiovascular_medical	0.0000				0.0000			
Respiratory medical	−0.0056	0.0330	−0.0107	− 0.0005	0.0038	0.4040	−0.0052	0.0128
Liver_GIS_medical	−0.0025	0.5190	−0.0100	0.0050	−0.0166	0.0120	−0.0296	−0.0036
CNS_medical	0.0123	0.0000	0.0063	0.0183	−0.0030	0.6480	−0.0158	0.0098
Sepsis	−0.0103	0.0000	−0.0156	−0.0050	− 0.0128	0.1330	− 0.0296	0.0039
Trauma	0.0050	0.2280	−0.0031	0.0132	−0.0213	0.0180	−0.0388	−0.0037
Metabolic Hormonal	0.0006	0.9110	−0.0107	0.0120	−0.1066	0.0000	−0.1178	−0.0955
Haematologic	−0.0065	0.4590	−0.0237	0.0107	0.0352	0.2230	−0.0214	0.0918
Renal_GUS	−0.0244	0.0000	−0.0349	−0.0140	− 0.0839	0.0000	− 0.0975	−0.0702
Other medical disorders	0.0142	0.3020	−0.0128	0.0413	−0.0472	0.0070	−0.0815	−0.0130
Musculoskeletal / Skin	−0.0074	0.5730	−0.0329	0.0182	−0.0583	0.0000	−0.0879	−0.0287
Cardio-Vascular surgery	−0.0006	0.9070	−0.0103	0.0091	−0.0710	0.0000	−0.0875	−0.0545
Thoracic surgery	0.0264	0.0070	0.0071	0.0457	−0.0536	0.0000	−0.0742	−0.0329
GIS surgery	−0.0015	0.6730	−0.0085	0.0055	−0.0481	0.0000	−0.0615	−0.0346
CNS surgery	0.0141	0.0150	0.0027	0.0254	0.0168	0.1070	−0.0036	0.0373
Traumatic/Orthopaedic surgery	0.0004	0.9470	−0.0107	0.0114	−0.0475	0.0000	−0.0664	−0.0286
Renal_GUS surgery	−0.0176	0.1000	−0.0387	0.0034	−0.0998	0.0000	−0.1148	−0.0848
Gynaecological	0.0834	0.0540	−0.0014	0.1681	−0.1142	0.0000	−0.1380	−0.0904
Musculoskeletal / Skin Surgery	0.0004	0.9530	−0.0138	0.0147	−0.0672	0.0000	−0.0814	−0.0530
Metabolic Surgery	0.0427	0.2800	−0.0348	0.1201	−0.0854	0.0000	−0.1286	−0.0421
Cardiovascular surgery elective	−0.0073	0.1330	−0.0168	0.0022	−0.1179	0.0000	−0.1302	−0.1056
Thoracic surgery elective	−0.0074	0.5570	−0.0320	0.0172	−0.0839	0.0000	−0.1056	−0.0622
GIS surgery elective	−0.0179	0.0040	−0.0300	−0.0059	− 0.0885	0.0000	− 0.0998	−0.0771
CNS surgery elective	0.0227	0.0980	−0.0042	0.0496	−0.0685	0.0000	−0.0916	−0.0453
Traumatic/Orthopaedic surgery el	−0.0345	0.1880	−0.0857	0.0168	−0.0946	0.0000	−0.1302	−0.0591
Renal_GUS surgery elective	0.0220	0.2250	−0.0135	0.0575	−0.0972	0.0000	−0.1185	−0.0760
Gynaecological surgery elective	−0.0491	0.1040	−0.1084	0.0101	−0.1264	0.0000	−0.1414	−0.1114
Musculoskeletal / Skin Surgery el	−0.0058	0.5760	−0.0262	0.0146	−0.0982	0.0000	−0.1151	−0.0813
Annual volume_deciles								
Base	0.0000				0.0000			
2	0.0016	0.6740	−0.0058	0.0090	0.0010	0.7740	−0.0058	0.0077
3	0.0092	0.0180	0.0016	0.0167	0.0064	0.0690	−0.0005	0.0134
4	0.0017	0.7270	−0.0080	0.0114	0.0085	0.1110	−0.0020	0.0190
5	−0.0008	0.8430	−0.0090	0.0074	0.0023	0.5740	−0.0057	0.0104
6	0.0047	0.2750	−0.0038	0.0133	0.0050	0.2680	−0.0038	0.0138
7	0.0032	0.4300	−0.0048	0.0112	0.0129	0.0040	0.0042	0.0217
8	0.0020	0.6310	−0.0063	0.0104	0.0066	0.1300	−0.0020	0.0152
9	−0.0030	0.5860	−0.0139	0.0078	−0.0042	0.4180	−0.0145	0.0060
10	−0.0047	0.2280	−0.0125	0.0030	−0.0022	0.6290	−0.0112	0.0068
Hospital classification								
Metropolitan	0.0000				0.0000			
Private	0.0145	0.0000	0.0089	0.0201	0.0124	0.0000	0.0071	0.0178
Rural / Regional	0.0034	0.2420	−0.0023	0.0092	0.0062	0.0260	0.0007	0.0117
Tertiary	0.0164	0.0000	0.0120	0.0209	0.0117	0.0010	0.0049	0.0184
Ventilation status								
Not-ventilated	0.0000				0.0000			
Ventilated	−0.0058	0.0010	−0.0093	−0.0023	0.0309	0.0710	−0.0026	0.0645

The IV paradigm is not without its limitations [21, 125] and has been subject to recent theoretical re-evaluation from within its archetypal domain, econometrics [126]. Such reviews have been relatively silent on the use of IV with binary outcome models [68, 125], although the LPM has been recommended [127]. The status of IV logistic regression, not implemented in current Stata™, was formally addressed by Foster in 1997 [128] using the Generalized Method of Moments, and more recently by two-stage residual inclusion estimation [129, 130], a preferred method in Mendelian randomisation [131], where identification of causal risk factors is the focus, rather than precise effect estimation [132]. This being said, two-stage residual inclusion has been shown to be a consistent estimator [129, 130]. IV logistic regression has seen implementation within the R statistical framework in the “naivreg” [133] and “ivtools” [134] packages.

## Conclusions

For modelling large scale binary outcome data, logistic regression, particularly the RE model, was the preferred estimator compared with probit and the LPM. The latter estimator cannot be recommended for probability generation. Endogeneity was demonstrated for hospital length of stay, risk of death and for MV treatment status. Accounting for endogeneity produced markedly different effect estimates about patient ventilation status compared with conventional methods. Exploration of and adjustment for endogeneity should be incorporated into modelling strategies, failure to do so may produce results that are “… less likely to be roughly right than they are to be precisely wrong” [120].

## Supplementary Information

**Additional file 1.**

## Data Availability

The dataset is the property of the ANZICS CORE and contributing ICUs and is not in the public domain. Access to the data by researchers, submitting ICUs, jurisdictional funding bodies and other interested parties is obtained under specific conditions and upon written request (“ANZICS CORE Data Access and Publication Policy.pdf”, http://www.anzics.com.au/Downloads/ANZICS%20CORE%20Data%20Access%20and%20Publication%20Policy%20July%202017.pdf).
