# Supplementary material for: Modelling hospital outcome: problems with endogeneity
Source: BMC Med Res Methodol. 2021 Jun 21;21:124. doi: 10.1186/s12874-021-01251-8 (PMC8215743; doi:10.1186/s12874-021-01251-8)
Supplement: Supplementary file 1 — Additional file 1. [file 12874_2021_1251_MOESM1_ESM.docx]

Appendix

Modelling hospital outcome: problems with endogeneity

Authors:

John L Moran, John D Santamaria , Graeme J Duke and the Australian & New Zealand Intensive Care Society (ANZICS) Centre for Outcomes & Resource Evaluation (CORE).

Base Logistic Model (estimate: odds ratio)

| Parameter | Estimate | P | Lower95%CI | Upper95%CI |
| --- | --- | --- | --- | --- |
| Age_centred | 1.042 | 0.000 | 1.033 | 1.052 |
| Age squared | 1.000 | 0.073 | 1.000 | 1.000 |
| APIII score_centred | 1.077 | 0.000 | 1.068 | 1.085 |
| APIII score squared | 1.000 | 0.000 | 1.000 | 1.000 |
| Gender | 1.000 |  | 1.000 | 1.000 |
| 1.gender | 1.005 | 0.874 | 0.949 | 1.064 |
| Age*APIII score | 1.000 | 0.000 | 1.000 | 1.000 |
| Cardiovascular_medical | 1.000 |  | 1.000 | 1.000 |
| Respiratory medical | 1.184 | 0.015 | 1.033 | 1.357 |
| Liver_GIS_medical | 0.635 | 0.000 | 0.502 | 0.802 |
| CNS_medical | 1.318 | 0.001 | 1.119 | 1.552 |
| Sepsis | 0.578 | 0.000 | 0.488 | 0.684 |
| Trauma | 0.571 | 0.000 | 0.441 | 0.740 |
| Metabolic Hormonal | 0.098 | 0.000 | 0.069 | 0.139 |
| Haematologic | 1.197 | 0.472 | 0.734 | 1.951 |
| Renal_GUS | 0.311 | 0.000 | 0.207 | 0.466 |
| Other medical disorders | 0.356 | 0.005 | 0.174 | 0.728 |
| Musculoskeletal / Skin | 0.250 | 0.008 | 0.090 | 0.699 |
| Cardio-Vascular surgery | 0.216 | 0.000 | 0.160 | 0.293 |
| Thoracic surgery | 0.349 | 0.000 | 0.225 | 0.542 |
| GIS surgery | 0.443 | 0.000 | 0.364 | 0.539 |
| CNS surgery | 1.525 | 0.000 | 1.215 | 1.914 |
| Traumatic/Orthopaedic surgery | 0.663 | 0.011 | 0.484 | 0.909 |
| Renal_GUS surgery | 0.073 | 0.000 | 0.028 | 0.189 |
| Gynaecological | 0.150 | 0.001 | 0.047 | 0.481 |
| Musculoskeletal / Skin Surgery | 0.311 | 0.000 | 0.213 | 0.455 |
| Metabolic Surgery | 0.159 | 0.107 | 0.017 | 1.488 |
| Cardiovascular surgery elective | 0.050 | 0.000 | 0.039 | 0.064 |
| Thoracic surgery elective | 0.199 | 0.000 | 0.140 | 0.284 |
| GIS surgery elective | 0.171 | 0.000 | 0.131 | 0.222 |
| CNS surgery elective | 0.176 | 0.000 | 0.123 | 0.251 |
| Traumatic/Orthopaedic surgery el | 0.141 | 0.024 | 0.026 | 0.775 |
| Renal_GUS surgery elective | 0.067 | 0.000 | 0.029 | 0.155 |
| Gynaecological surgery elective | 0.025 | 0.000 | 0.003 | 0.193 |
| Musculoskeletal / Skin Surgery el | 0.093 | 0.000 | 0.060 | 0.144 |
| APIII categories#APIII score | 1.000 |  | 1.000 | 1.000 |
| 1b.ap3diagnosis2#co.c_ap3score | 1.000 |  | 1.000 | 1.000 |
| 2.ap3diagnosis2#c.c_ap3score | 0.988 | 0.000 | 0.984 | 0.992 |
| 3.ap3diagnosis2#c.c_ap3score | 1.007 | 0.019 | 1.001 | 1.013 |
| 4.ap3diagnosis2#c.c_ap3score | 0.997 | 0.112 | 0.992 | 1.001 |
| 5.ap3diagnosis2#c.c_ap3score | 1.001 | 0.474 | 0.998 | 1.005 |
| 6.ap3diagnosis2#c.c_ap3score | 1.018 | 0.000 | 1.011 | 1.026 |
| 7.ap3diagnosis2#c.c_ap3score | 1.007 | 0.077 | 0.999 | 1.014 |
| 8.ap3diagnosis2#c.c_ap3score | 0.993 | 0.247 | 0.980 | 1.005 |
| 9.ap3diagnosis2#c.c_ap3score | 0.995 | 0.297 | 0.985 | 1.005 |
| 10.ap3diagnosis2#c.c_ap3score | 1.008 | 0.441 | 0.988 | 1.029 |
| 11.ap3diagnosis2#c.c_ap3score | 1.027 | 0.119 | 0.993 | 1.061 |
| 12.ap3diagnosis2#c.c_ap3score | 1.019 | 0.000 | 1.011 | 1.027 |
| 13.ap3diagnosis2#c.c_ap3score | 1.010 | 0.201 | 0.995 | 1.026 |
| 14.ap3diagnosis2#c.c_ap3score | 1.003 | 0.234 | 0.998 | 1.008 |
| 15.ap3diagnosis2#c.c_ap3score | 0.999 | 0.809 | 0.991 | 1.007 |
| 16.ap3diagnosis2#c.c_ap3score | 1.008 | 0.064 | 1.000 | 1.017 |
| 17.ap3diagnosis2#c.c_ap3score | 1.019 | 0.078 | 0.998 | 1.040 |
| 18.ap3diagnosis2#c.c_ap3score | 1.001 | 0.955 | 0.959 | 1.046 |
| 19.ap3diagnosis2#c.c_ap3score | 1.009 | 0.105 | 0.998 | 1.020 |
| 22.ap3diagnosis2#c.c_ap3score | 1.043 | 0.267 | 0.968 | 1.124 |
| 23.ap3diagnosis2#c.c_ap3score | 1.021 | 0.000 | 1.014 | 1.029 |
| 24.ap3diagnosis2#c.c_ap3score | 0.996 | 0.675 | 0.979 | 1.014 |
| 25.ap3diagnosis2#c.c_ap3score | 1.006 | 0.190 | 0.997 | 1.015 |
| 26.ap3diagnosis2#c.c_ap3score | 1.025 | 0.009 | 1.006 | 1.044 |
| 27.ap3diagnosis2#c.c_ap3score | 1.000 | 0.992 | 0.951 | 1.051 |
| 28.ap3diagnosis2#c.c_ap3score | 1.034 | 0.046 | 1.001 | 1.068 |
| 29.ap3diagnosis2#c.c_ap3score | 1.009 | 0.854 | 0.915 | 1.113 |
| 30.ap3diagnosis2#c.c_ap3score | 1.026 | 0.007 | 1.007 | 1.045 |
| Annual volume deciles | 1.000 |  | 1.000 | 1.000 |
| 2.annvol_deciles | 1.019 | 0.861 | 0.822 | 1.265 |
| 3.annvol_deciles | 1.046 | 0.683 | 0.843 | 1.298 |
| 4.annvol_deciles | 1.147 | 0.325 | 0.873 | 1.509 |
| 5.annvol_deciles | 1.058 | 0.633 | 0.838 | 1.337 |
| 6.annvol_deciles | 1.069 | 0.581 | 0.844 | 1.354 |
| 7.annvol_deciles | 1.082 | 0.492 | 0.864 | 1.357 |
| 8.annvol_deciles | 0.974 | 0.829 | 0.770 | 1.233 |
| 9.annvol_deciles | 0.997 | 0.984 | 0.737 | 1.349 |
| 10.annvol_deciles | 0.844 | 0.131 | 0.677 | 1.052 |
| Annual volume deciles#APIII | 1.000 |  | 1.000 | 1.000 |
| 2.annvol_deciles#c.c_ap3score | 1.001 | 0.593 | 0.996 | 1.007 |
| 3.annvol_deciles#c.c_ap3score | 1.003 | 0.257 | 0.998 | 1.008 |
| 4.annvol_deciles#c.c_ap3score | 0.999 | 0.819 | 0.992 | 1.006 |
| 5.annvol_deciles#c.c_ap3score | 1.002 | 0.457 | 0.996 | 1.008 |
| 6.annvol_deciles#c.c_ap3score | 1.001 | 0.695 | 0.995 | 1.007 |
| 7.annvol_deciles#c.c_ap3score | 1.005 | 0.120 | 0.999 | 1.010 |
| 8.annvol_deciles#c.c_ap3score | 1.006 | 0.072 | 1.000 | 1.012 |
| 9.annvol_deciles#c.c_ap3score | 1.004 | 0.326 | 0.996 | 1.012 |
| 10.annvol_deciles#c.c_ap3score | 1.006 | 0.026 | 1.001 | 1.012 |
| Annual volume deciles#Age | 1.000 |  | 1.000 | 1.000 |
| 2.annvol_deciles#c.c_age | 1.000 | 0.936 | 0.991 | 1.009 |
| 3.annvol_deciles#c.c_age | 0.998 | 0.577 | 0.989 | 1.006 |
| 4.annvol_deciles#c.c_age | 0.989 | 0.040 | 0.978 | 0.999 |
| 5.annvol_deciles#c.c_age | 0.990 | 0.040 | 0.981 | 1.000 |
| 6.annvol_deciles#c.c_age | 0.990 | 0.028 | 0.981 | 0.999 |
| 7.annvol_deciles#c.c_age | 0.989 | 0.011 | 0.980 | 0.997 |
| 8.annvol_deciles#c.c_age | 0.989 | 0.015 | 0.980 | 0.998 |
| 9.annvol_deciles#c.c_age | 0.981 | 0.002 | 0.969 | 0.993 |
| 10.annvol_deciles#c.c_age | 0.986 | 0.001 | 0.979 | 0.994 |
| Hospital classification: Metropolitan | 1.000 |  | 1.000 | 1.000 |
| Private | 1.096 | 0.231 | 0.943 | 1.273 |
| Rural / Regional | 1.202 | 0.038 | 1.010 | 1.431 |
| Tertiary | 1.615 | 0.000 | 1.418 | 1.840 |
| Ventilation status | 1.000 |  | 1.000 | 1.000 |
| Ventilated | 1.287 | 0.000 | 1.169 | 1.418 |
| Hospital level#APIII score | 1.000 |  | 1.000 | 1.000 |
| 2.hoslevel#c.c_ap3score | 1.005 | 0.045 | 1.000 | 1.009 |
| 3.hoslevel#c.c_ap3score | 1.000 | 0.928 | 0.995 | 1.004 |
| 4.hoslevel#c.c_ap3score | 0.997 | 0.132 | 0.994 | 1.001 |
| Ventialtion#APIII score | 1.000 |  | 1.000 | 1.000 |
| 1.ventilated#c.c_ap3score | 0.991 | 0.000 | 0.988 | 0.993 |
| Constant | 0.125 | 0.000 | 0.091 | 0.172 |

#, interaction. 1.gender, male (vs female) c.c_ap3score, centred APACHE III score specified as a continuous variable in Stata (c.). APIII, centred APACHE III. ap3diagnosis2, consolidated APACHE III diagnostic codes (see below). Annual volume deciles; patient annual admission volume

Consolidated APACHE III Diagnostic Codes

| APACHE III diagnostic codes | Freq. | Percent | Cum. |
| --- | --- | --- | --- |
|  |  |  |  |
| Cardiovascular_medical | 6,991 | 7.54 | 7.54 |
| Respiratory medical | 8,945 | 9.65 | 17.19 |
| Liver_GIS_medical | 2,369 | 2.56 | 19.75 |
| CNS_medical | 4,708 | 5.08 | 24.83 |
| Sepsis | 6,202 | 6.69 | 31.52 |
| Trauma | 2,677 | 2.89 | 34.41 |
| Metabolic Hormonal | 5,929 | 6.40 | 40.80 |
| Haematologic | 311 | 0.34 | 41.14 |
| Renal_GUS | 1,326 | 1.43 | 42.57 |
| Other medical disorders | 457 | 0.49 | 43.06 |
| Musculoskeletal / Skin | 226 | 0.24 | 43.31 |
| Cardio-Vascular surgery | 2,220 | 2.40 | 45.70 |
| Thoracic surgery | 935 | 1.01 | 46.71 |
| GIS surgery | 4,242 | 4.58 | 51.29 |
| CNS surgery | 1,376 | 1.48 | 52.77 |
| Traumatic/Orthopaedic surgery | 1,263 | 1.36 | 54.13 |
| Renal_GUS surgery | 554 | 0.60 | 54.73 |
| Gynaecological | 602 | 0.65 | 55.38 |
| Musculoskeletal / Skin Surgery | 1,313 | 1.42 | 56.80 |
| Metabolic Surgery | 100 | 0.11 | 56.90 |
| Cardiovascular surgery elective | 17,335 | 18.70 | 75.61 |
| Thoracic surgery elective | 3,596 | 3.88 | 79.48 |
| GIS surgery elective | 6,636 | 7.16 | 86.64 |
| CNS surgery elective | 6,012 | 6.49 | 93.13 |
| Traumatic/Orthopaedic surgery elective | 134 | 0.14 | 93.27 |
| Renal_GUS surgery elective | 1,330 | 1.43 | 94.71 |
| Gynaecological surgery elective | 817 | 0.88 | 95.59 |
| Musculoskeletal / Skin Surgery elective | 4,087 | 4.41 | 100.00 |
|  |  |  |  |
| Total | 92,693 | 100.00 |  |
